# Management of preoperative pain in elderly patients with moderate to severe cognitive deficits and hip fracture: a retrospective, monocentric study in an orthogeriatric unit

**DOI:** 10.1186/s12877-021-02500-7

**Published:** 2021-10-19

**Authors:** Mathilde Ruel, Bastien Boussat, Mehdi Boudissa, Virginie Garnier, Catherine Bioteau, Jérôme Tonetti, Régis Pailhe, Gaëtan Gavazzi, Sabine Drevet

**Affiliations:** 1 Orthogeriatric Unit, University Hospital Grenoble Alpes, Grenoble, France; 2 Public Health Department, University Hospital Grenoble Alpes, Grenoble, France; 3 Orthopaedic and Traumatology Surgery Department, University Hospital Grenoble Alpes, Grenoble, France; 4 Geriatric Department, University Hospital Grenoble Alpes, Grenoble, France; 5 Rocheplane Rehabilitation Center, Grenoble, France

**Keywords:** Elderly, Cognitive deficits, Hip fracture, Preoperative, Pain, Opioids, Orthogeriatric

## Abstract

**Background:**

Patients with cognitive deficits are 3 times more likely to suffer a hip fracture than geriatric patients of the same age group without cognitive deficits. The persistence of perioperative pain following hip fracture is a risk factor for the occurrence of delirium, poor functional prognosis, and the development of secondary chronic pain. Patients with cognitive deficits receive 20 to 60% less analgesics than those without cognitive deficits. Our retrospective descriptive monocentric study was performed in an orthogeriatric unit on a cohort of elderly patients hospitalized for hip fracture. The aim of the study was to compare the quantity of strong opioids delivered in a morphine sulfate equivalent daily during the preoperative period after a hip fracture between cognitively intact patients and those with cognitive deficits.

**Results:**

Our total population of 69 patients had a median age of 90 years old, and 46% of these patients had moderate or severe cognitive deficits. During the preoperative period, the same quantity of strong opioids was administered to both groups of patients (13.1 mg/d versus 10.8 mg/d (*p* = 0.38)). Patients with moderate to severe cognitive deficits more often experienced delirium during their hospitalization (*p* < 0.01) and received more psychotropic drugs in the first 3 postoperative days (*p* = 0.025).

**Conclusions:**

We reported that with standardized pain management in an orthogeriatric unit, patients aged 75 years and older received the same daily average quantity of strong opioids during the preoperative period regardless of the presence of cognitive deficits.

## Introduction

Hip fracture is one of the most serious consequences of falls in elderly subjects [1]. The hip fracture risk is 9 to 19% higher for the population over 80 years old [2, 3]. Patients with cognitive deficits are 3 times more likely to suffer a hip fracture than a comparable age group of patients without cognitive deficits [4]. This increasing risk of fracture can be explained by an increased risk of fall linked to multiple reasons, including the impairment of executive functions, agnosia disorders and the impairment of instrumental functions. In addition, frequent prescription of psychotropic drugs in this patient population is a risk factor [5]. Regardless of cognitive status, mortality rates during the first postfracture year vary from 12 to 35% depending on different studies [3, 6, 7]. A high excess mortality risk from all causes is noted during the first months following fracture and predominates in the male population [2, 6, 8]. Postfracture morbidity is high with an increased risk of regarding cardiovascular events, pulmonary infections or depressive events [3, 9]. Hip fracture also has a major impact on functional status in elderly patients. One year after a hip fracture, 20% of patients lost their walking capacity, 30 to 50% showed partial dependency, and 30% of cases showed full dependency [3, 10, 11].

Older subjects with multimorbid conditions also suffer acute postfracture pain in addition to existing chronic pain [12]. Acute pain is mainly due to nociception excess with frequent neuropathic pain involvement [13]. The persistence of perioperative pain following hip fracture is a risk factor for the occurrence of delirium [14], poor functional prognosis [15–17], and the development of secondary chronic pain [18]. Additionally, preoperative delay is described as a risk factor influencing the intensity of postoperative pain [19]. Guidelines recommend multimodal analgesia that can combine nonopioid treatments (acetaminophen systematically), strong opioids if the intensity of pain requires this [13] and locoregional anaesthesia (femoral or iliofascial block) [13, 20]. Analgesic management also combines pain assessment and nondrug proposals [12]. However, the traction method is not recommended as part of analgesic management [11, 21].

Frequent atypical clinical presentations in older subjects may explain why the evaluation of pain for elderly patients may be more complicated compared with that for young adults. Furthermore, patients with cognitive deficits can present communication disorders that make evaluation of pain even more difficult [22–24]. Several comparative studies highlight that postoperative prescriptions to patients with cognitive deficits include between 20 and 60% less analgesics than that for cognitively intact patients [25–29]. Preoperative pain is also believed to be treated less often in patients with cognitive deficits [27, 28]. To our knowledge, the management of analgesic drugs in the preoperative period of hip fracture is unknown, particularly for patients 75 years old and over. We hypothesize that geriatric patients with moderate or severe cognitive deficits receive fewer strong opioids than other geriatric patients with no or mild cognitive deficits. Here, the aim of our study was to compare preoperative analgesic management of hip fracture in patients aged 75 years and older with or without cognitive deficits.

## Materials and methods

This was a retrospective descriptive monocentric study. Our consecutive patient cohort consisted of subjects aged 75 years old and older hospitalized for hip fracture in the orthogeriatric units of the Grenoble Alpes University Hospital (France) located in the orthopaedic and traumatology surgery department. The inclusion period was from 09 october 2018 to 13 may 2019. The study followed CNIL (National Commission on Informatics and Liberty) and RGPD (General Data Protection Regulation) recommendations. Study registration within the internal register for processing activities of the Data Protection Officer (DPO) controller was performed prior to Clinical Research and Innovation Delegation (DRCI) approval. Patients and their families were informed about the study and could refuse to participate.

**Fig. 1:**
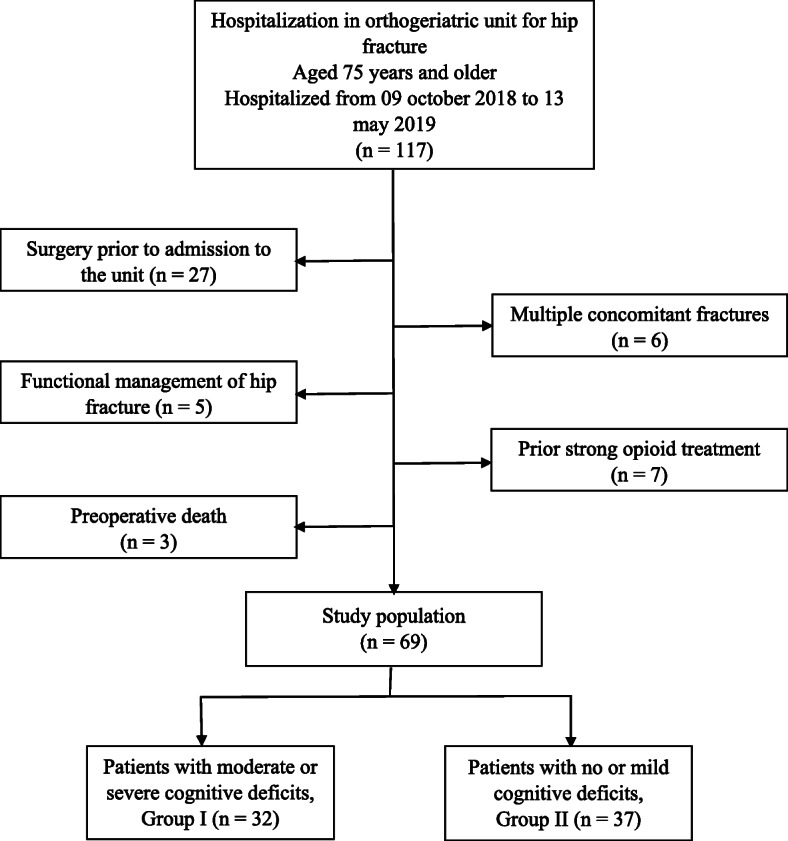
Flow chart

The exclusion criteria were surgery prior to admission to the unit, functional management of hip fracture, and preoperative death. To avoid interpretation bias, we excluded patients with multiple concomitant fractures and patients who previously had strong opioid treatment (usual treatment) (Fig. 1).

The aim of our study was to compare preoperative analgesic management of hip fracture in patients 75 years old and older with or without moderate to severe cognitive deficits. The main critical criterion was the average quantity of strong opioids delivered in a morphine sulfate equivalent in milligrams per day (mg/d). Equivalence calculations for strong opioids were performed according to the equianalgesic Table [30].

Our secondary aims were to compare the 2 groups of patients over various time periods based on the following criteria: 1) pain assessment using numerical scales and the quantity of acetaminophen delivered in grams per day (g/d) in the preoperative period and the first 3 postoperative days; 2) the quantity of strong opioids delivered in a morphine sulfate equivalent in mg/d in the first 3 postoperative days; 3) the occurrence of delirium during hospitalization according to the scale of the Confusion Assessment Method (CAM) [31]; and 4) administration or absence of psychotropics in the preoperative period and the first 3 postoperative days.

The following data were collected: gender, age, type of fracture and of surgery, preoperative delay in hours, length of stay in days, living place, pre-fracture functional status according to Katz’s Activities of Daily Living scale (ADL) [32] and Lawton’s Instrumental Activities of Daily Living scale (IADL) [33], walking ability with or without technical assistance, the presence or absence of severe renal failure (glomerular filtration rate less than 30 ml/min), weight in kilograms, pain assessment using numerical scale rated out of 10, the quantity of strong opioids delivered in a morphine sulfate equivalent in mg/d, the quantity of acetaminophen delivered in g/d, long-term treatment with psychotropic drugs (usual treatment) (benzodiazepines, such as lorazepram, oxazepram, prazepam, bromazepam, and alprazolam; neuroleptics, including risperidone; and hypnotic drugs, such as zolpidem and zopiclone), the administration of psychotropic drugs, the occurrence of delirium during hospitalization, the comorbidities score according to the Cumulative Illness Rating Scale – Geriatric (CIRS-G) assessed based on 56 points [34], the Charlson index score assessed by 24 points [35], and the American Society of Anaesthesiologists score (ASA) assessed based on 6 points [36].

In order to distinguish our two groups according the cognitive status, we considered the MMSE less than 6 months old previous the hip fracture to determine if the patient were in one or the other group. Geriatric patients with moderate or severe cognitive deficits (group I) were defined according to the known MMSE with a score below 20/30 regardless of the aetiology of the disorder. If the MMSE score was greater than 21/30 or not available, the patient was included in group II. Patients with MMSE scores between 21 and 25/30 were not considered patients with moderate or severe cognitive deficits because their verbalization, understanding, and participation in pain self-assessment would be less affected within this population [22, 24].

The orthogeriatric unit employs a set of protocols and standardized pain management (SPM) that consists of the following: 1) pain assessment through a numerical scale performed systematically 3 times a day and then repeated as many times as necessary. The numerical scale is a self-assessment scale with a maximum score of 10 where 0 indicates no pain and 10 indicates unbearable pain; the algoplus scale was performed in addition in case of severe cognitive deficits 2) non-pharmacological pain management included during the preoperative period: the limitation of the movements of the traumatized limb by positioning in alignment of the limb, blocking rotations by avoiding muscular contractions of the traumatized limb, mobilization in the block by trained paramedical teams, and ice during the peri-operative period; 3) systematic prescription of 1 g of acetaminophen 3 times a day and 5 mg per os of oxycodone (or equivalent) systematically delivered in the morning before the nursing and mobilization procedures; 4) conditional prescription of strong opioids based on pain intensity all day. A pain intensity level greater than 6 induces opioid use. Data tracking of the numerical scale of prescriptions and analgesics given was performed by nursing staff.

Study data were collected through patient electronic records using Cristalnet and Easily software. Descriptive analysis was conducted on all of the variables collected, on the total population collected, and in each group according to the categorical variable defined as the presence or absence of cognitive deficits. Qualitative parameters were expressed in numbers and percentages. Quantitative parameters were described by the mean ± standard deviation and by median with the 25th and 75th percentiles. Descriptive analysis of the different variables according to the categorical variable was analysed by univariate tests. Quantitative data were compared using the Mann-Whitney test or Welch test, and qualitative data were compared using the Chi-2 test or nonparametric Fisher test. No multivariate analysis was performed due to the low number of patients involved in our study. Analysis was performed using data processed in Excel 2019 for PC, and statistics were performed on Pvalue.io. A *p*-value less than 0.05 indicated a significant result.

## Results

Among the 117 patients admitted for hip fracture within orthogeriatric units, 69 patients were included in our panel after applying our exclusion criteria (Fig. 1).

The population characteristics are presented in Table 1. The average age was 89 years old, and the median age was 90 years old. The female gender represented 78% of the total population. Thirty-two patients (46%) presented moderate or severe cognitive deficits before hip fracture (group I). Compared to the patients with no or mild cognitive deficits (group II), group I was more dependent with an average ADL score of 2.89/6 versus 4.74/6 (*p* < 0.001) and an average IADL score of 0.48/8 versus 3.65/8 (*p <* 0.001). Patients in this group also lived more frequently in nursing homes (*p <* 0.001). The weight and presence of severe renal failure were comparable in both groups (Table 1).

**Table 1:** Population characteristics

	Total population	Geriatric patients with moderate or severe cognitive deficits (Group I)	Geriatric patients with no or mild cognitive deficits (Group II)	P
**Population**				
Number	**69 (100)**	**32 (46)**	**37 (54)**	
Gender, female	54 (78)	26 (81)	28 (76)	0.58
Age, years	89.0 (+/−5.17) / 90.0 [86.0–92.0]	89.1 (+/−5.18)	88.9 (+/−5.22)	0.87
**Surgery**				
Type of fracture				
Femoral neck	39 (57)	16 (50)	23 (62)	0.63
Pertrochanteric	26 (38)	14 (44)	12 (32)	–
Peri-prosthetic	4 (5.8)	2 (6.2)	2 (5.4)	–
Preoperative delay, hours	80.4 (+/−50.6) / 72.0 [48.0–96.0]	78.0 (+/− 51.1)	82.3 (+/− 50.9)	0.72
Type of surgery				
Total hip prosthesis	9 (13)	2 (6.2)	7 (19)	0.46
Intermediate hip prosthesis	30 (43)	15 (47)	15 (41)	–
Gamma nail osteosynthesis	27 (39)	14 (44)	13 (35)	–
Plate Osteosynthesis	3 (4.3)	1 (3.1)	2 (5.4)	–
Length of stay, days	15.5 (+/−6.13) / 14.0 [12.0–18.0]	14.8 (+/−5.99)	16.1 (+/−6.25)	0.36
**Pre-fracture functional status and living place**				
Pre-fracture functional status				
ADL (/6)	3.91 (+/−1.77) / 4.00 [2.75–5.50]	2.89 (+/−1.44)	4.74 (+/−1.58)	**< 0.001**
IADL (/8)	2.26 (+/− 2.80) / 1.00 [0–5.00]	0.48 (0.83)	3.65 (+/− 3.01)	**< 0.001**
Walking ability with or without technical assistance	53 (82)	21 (72)	32 (89)	0.089
Living place				
Community facility	39 (57)	10 (31)	29 (78)	**< 0.001**
Nursing home	30 (43)	22 (69)	8 (22)	**–**
**Comorbidities**				
Moderate or severe cognitive deficits	32 (46)	100 (100)	0 (0)	
Weight, kilograms	57.5 (+/−14.2) / 55.0 [47.0–66.0]	57.2 (+/−11.8)	57.7 (+/− 16.0)	0.89
Severe renal failure (GFR Cockcroft < 30 ml/min)	11 (16)	3 (9.4)	8 (22)	0.17
Scales				
CIRS-G^a^ (/56)	10.0 (+/−2.93) / 10.0 [8.00–12.00]	10.1 (+/−2.60)	9.96 (+/−3.24)	0.89
ASA (/6)	2.86 (+/−0.661) / 3.00 [2.25–3.00]	3.00 (+/−0.620)	2.74 (+/−0.682)	0.16
Charlson^a^ (/24)	3.40 (+/−2.39) / 3.00 [2.00–4.00]	3.54 (+/−1.89)	3.29 (+/−2.79)	0.38

Data are expressed as numbers (%), means (+/− standard deviation), or medians [25–75 percentile]

^a^20–25% Missing data

Regarding the total population of our study, the quantity of strong opioids delivered in a morphine sulfate equivalent daily was on average 11.9 (+/− 10.7) mg/d in the preoperative period. Patients in group I received an average of 13.1 (+/− 9.20) mg/d, and patients in group II received 10.8 (+/− 11.9) mg/d. No significant difference between these groups was identified (*p* = 0.38) (Table 2).

**Table 2:** Results

	Total population	Geriatric patients with moderate or severe cognitive deficits (Group I)	Geriatric patients with no or mild cognitive deficits (Group II)	P
**Quantity of strong opioids administered in the preoperative period**				
Quantity of strong opioids administered (in morphine sulfate equivalent, mg/d)	11.9 (+/− 10.7) / 10.0 [5.00–17.8]	13.1 (+/−9.20)	10.8 (+/−11.9)	0.38
**Pain assessment and quantity of paracetamol administered in the preoperative period**				
Average Numerical Scale (/10)	1.34 (+/−1.12) / 1.15 [0.675–1.80]	1.25 (+/− 0.906)	1.41 (+/−1.27)	0.97
Maximum Numerical Scale (/10)	3.91 (+/−2.44) / 4.00 [2.00–5.00]	3.79 (+/−2.13)	4.00 (+/−2.68)	0.86
Quantity of paracetamol (g/d)	1.76 (+/−0.907) / 1.80 [1.25–2.33]	1.78 (+/−0.880)	1.73 (+/−0.941)	0.83
**Pain assessment, quantity of paracetamol administered, and quantity of strong opioids administered in the first 3 postoperative days**				
Average Numerical Scale (/10)	1.04 (+/−0.663) / 0.900 [0.600–1.35]	1.03 (+/−0.704)	1.06 (+/−0.636)	0.89
Maximum Numerical Scale (/10)	3.61 (+/−1.87) / 3.00 [2.00–5.00]	3.35 (+/−1.94)	3.83 (+/−1.80)	0.3
Quantity of paracetamol (g/d)	2.54 (+/−2.08) / 2.00 [1.20–3.33]	2.49 (+/−2.07)	2.59 (+/−2.11)	0.84
Quantity of strong opioids administered (in morphine sulfate equivalent, mg/d)	14.5 (+/−13.3) / 10.0 [6.67–16.7]	16.8 (+/−14.9)	12.5 (+/−11.5)	0.19
**Delirium and psychotropic drugs**				
Occurrence of delirium during hospitalization	16 (24)	12 (39)	4 (11)	**< 0.01**
Long term psychotropic drugs treatment (usual treatment)	23 (33)	15 (47)	8 (22)	**0.026**
Psychotropic drugs administered in preoperative period	36 (52)	20 (62)	16 (43)	0.11
Psychotropic drugs administered in the first 3 postoperative days	31 (45)	19 (59)	12 (32)	**0.025**

Data are expressed as numbers (%), means (+/− standard deviation), or medians [25–75 percentile]

Regarding secondary objectives, pain assessment using the numerical scale during the preoperative period was comparable between the 2 groups with an average of 1.25/10 for patients in group I and an average of 1.41/10 for patients in group II (*p* = 0.97). In the preoperative period, the quantity of acetaminophen delivered daily was equivalent for both groups with an average of 1.78 g/d for group I and 1.73 g/d for group II (*p* = 0.83). During the first 3 postoperative days, no differences were noted between the 2 groups regarding pain assessment (*p* = 0.89), quantity of acetaminophen delivered (*p* = 0.84), or the quantity of strong opioids delivered (*p* = 0.19) (Table 2). The occurrence of delirium during hospitalization was more frequent for patients with severe cognitive deficits with 39% of these patients exhibiting delirium compared to 11% for patients with no cognitive deficits (*p* < 0.01). Regarding patients’ usual treatments, prescription for patients with moderate or severe cognitive deficits included more psychotropic drugs than patients with no or mild cognitive deficits (*p* = 0.026). In the preoperative period, psychotropic drugs were delivered in a comparable manner (62% of patients with severe cognitive deficits versus 43% of patients with no or mild cognitive deficits, *p* = 0.11). During the first 3 postoperative days, psychotropic drugs were delivered more frequently to patients with moderate or severe cognitive deficits (59%) compared with patients with no or mild cognitive deficits (32%) (*p* = 0.025) (Table 2).

If we compared patients based on delirium rather than on cognitive deficits, the daily average quantity of strong opioids delivered was comparable between patients with or without cognitive deficits in the preoperative period (*p* = 0.71) and during the first 3 postoperative days (*p* = 0.57) (Table 3).

**Table 3:** Quantity of analgesics administered to patients with or without delirium

	Patients without delirium	Patients with delirium	P
Occurrence of delirium during hospitalization	0 (0)	100 (100)	
**Pain assessment, quantity of paracetamol administered, and quantity of strong opioids administered in preoperative period**			
Average Numerical Scale (/10)	1.43(+/−1.20)	1.07(+/−0.809)	0.56
Maximum Numerical Scale (/10)	4.17(+/−2.39)	3.12(+/−2.50)	0.18
Quantity of paracetamol (g/d)	1.73(+/−0.971)	1.79(+/−0.678)	0.86
Quantity of strong opioids administered (in morphine sulfate equivalent, mg/d)	11.7(+/−11.1)	12.2(+/−9.94)	0.71
**Pain assessment, quantity of paracetamol administered, and quantity of strong opioids administered in the first 3 postoperative days**			
Average Numerical Scale (/10)	1.08(+/−0.688)	0.925(+/−0.580)	0.38
Maximum Numerical Scale (/10)	3.67(+/−1.90)	3.44(+/− 1.79)	0.52
Quantity of paracetamol (g/d)	2.46(+/−2.00)	2.82(+/− 2.44)	0.97
Quantity of strong opioids administered (in morphine sulfate equivalent, mg/d)	13.4(+/−11.4)	18.4(+/−18.2)	0.57

Data are expressed as numbers (%), means (+/− standard deviation), or medians [25–75 percentile]

*mg/d* milligram per day, *g/d* gram per day

## Discussion

Our study of the preoperative period of a hip fracture showed that with standardized pain management in an orthogeriatric unit, patients with or without cognitive deficits received the same daily average quantity of strong opioids. The pain rate and the quantity of acetaminophen delivered did not differ between patient groups on the preoperative or the first 3 postoperative days. However, patients with severe cognitive deficits more often suffered from delirium during hospitalization and more frequently received psychotropic drugs during the first 3 postoperative days.

Our results were different from those reported in the literature [25–29]. In our study, we highlighted the fact that the cognitive status of patients aged 75 years old and older who are hospitalized in orthogeriatric units for hip fracture does not impact the quantity of analgesics delivered during the preoperative period.

This study was performed within a perioperative orthogeriatric unit, which provides more overall care for elderly patients than a conventional orthopaedic surgery unit. Indeed, medical-surgical care within the orthogeriatric unit offers global, multidisciplinary services (geriatrician, surgeon, anaesthetist) [11, 37]. Standardized pain management in the perioperative orthogeriatric unit could explain the homogeneity of care and therefore our results. An SPM can help to optimize the pain management of patients. Nevertheless, the protocol remains weak, and the quality of the management and the training of the team and the geriatric culture must be optimized to ensure effective care.

Cognitive comorbidity is often undiagnosed by community players, and patients could have physiological cognitive dysfunctions associated with their age [38]. When forming the two groups, there was a potential risk of ignoring the presence of mild cognitive deficits, but moderate or severe cognitive deficit diagnosis in an acute traumatic context is neither recommended nor well adapted. The definition of severe cognitive deficits is extremely heterogeneous among studies [25, 28, 29]. The MMSE score is a screening assessment for cognitive impairment but not a diagnostic tool for dementia. On the other hand, the use of the MMSE is not recommended to screen cases of cognitive deficits during the acute phase. Thus, we discuss the real possibility of defining study groups based on cognitive criteria in a traumatological emergency context. There is a high risk of incorrectly concluding moderate or severe cognitive deficits in the perioperative period due to the high prevalence of delirium [39]. The occurrence of perioperative delirium may be a sign of cognitive vulnerability and can suggest underlying undiagnosed cognitive deficits [40]. Delirium should not be confused with the presence of cognitive deficits, and a second evaluation should be made independently of the acute traumatological event.

Delirium is one of the most frequent perioperative complications of hip fracture, and its incidence is 2 to 3 times higher for patients with dementia [39, 41]. In our study, the occurrence of delirium during hospitalization was more frequently noted in patients with dementia. The presence of delirium makes pain assessment more difficult. Overall, pain syndrome is underestimated and underdiagnosed in elderly subjects because pain assessment can be difficult to establish, especially [42] for patients with cognitive deficits [22–24]. Capacities to express, understand and participate in pain self-assessment can be impaired. The SPM content used only a numerical scale to compare both groups. The pain rate using the numerical scale provided a low score for both groups. The numerical scale (self-assessment scale) used for all patients could have been supplemented by the use of the Algoplus scale, which has been validated and recommended for “noncommunicative” patients [22, 24, 43]. Based on our results, we expected that the quantity of strong opioids administered to patients with delirium would be lower than the quantity administered to patients without delirium. However, our analysis based on the confusion categorical variable showed that the quantity of strong opioids delivered was similar to both groups with and without delirium. Therefore, regardless of the presence of delirium or cognitive deficits, patients received similar quantities of analgesics for hip fracture care within an orthogeriatric unit.

A link was established between untreated or inadequately treated perioperative pain and the occurrence of confusion [14, 44]. This finding can be explained by the following “Bouchon’s 1 + 2 + 3” pattern [45], whereby perioperative pain is assimilated to acute stress, which can lead to or participate in organ function decompensation, which is already weakened by the ageing process and the presence of a chronic pathology, namely, a cognitive pathology. For an elderly person with impaired brain capacity, pain stress can lead to the occurrence of delirium. Thus, a prolonged preoperative delay is a delirium risk factor, especially for patients with severe cognitive deficits [46]. In our study, the preoperative delay before surgery was long regardless of the patient’s cognitive status, but our main judging criteria in the preoperative period required the exclusion of patients with a short preoperative delay. The determinants of the delay are related to the patient and his clinical status as well as institutional and organizational conditions. In summary, delirium can also be a side effect of strong opioids or psychotropic drugs [47, 48]. The pharmacokinetics and pharmacodynamics of these drugs are modified due to the ageing process and result in a high sensitivity of therapeutics; therefore, these drugs need to be used with caution in elderly subjects [12, 22]. One-third of our study population underwent long-term psychotropic drug treatment before their hip fracture, and patients with cognitive deficits took it more often than those without. Their treatment was adjusted to their usual psychotropic treatment at the beginning of their hospitalization. At the end of this preoperative drug conciliation, the psychotropic drugs administered were similar for both groups. A new difference was highlighted in the postoperative period, during which psychotropic drugs were administered more frequently to patients with severe cognitive deficits. The timeline between the occurrence of delirium and the administration of psychotropic drugs was not indicated; thus, it was difficult to link causality between delirium and psychotropic drugs.

Our study had several limitations. This study was retrospective and monocentric. However, our study included patients who were older than those previously included the literature with a median age of 90 years [25]. The characteristics of both groups were comparable, making our cohort homogeneous. The objectified differences in functional status and living location were expected for patients with severe cognitive deficits who by definition were more dependent on these factors [49].

Our study demonstrated that regardless of the presence of cognitive deficits and delirium, patients 75 years old and over hospitalized in the orthogeriatric unit for a hip fracture received the same daily average quantity of strong opioids during the preoperative period. Standard pain management in an orthogeriatric unit can avoid the undertreatment of pain in patients with moderate to strong cognitive deficits. The expertise and the quality of multidisciplinary care in our perioperative orthogeriatric unit could explain the homogeneity of care. Indeed, orthogeriatric care pathways are recommended to improve the prognosis of these patients when hospitalized for hip fracture [11, 37].

## Data Availability

The datasets generated and analysed during the current study are not publicly available in the absence of a confidentiality agreement but are available from the Dr. S. Drevet author on reasonable request (SDrevet@chu-grenoble.fr) and after consultation with the institution’s legal service.

## References

[1] Cuevas-Trisan R (2017). Balance problems and fall risks in the elderly. Phys Med Rehabil Clin N Am.

[2] Bliuc D, Nguyen ND, Milch VE, Nguyen TV, Eisman JA, Center JR (2009). Mortality risk associated with low-trauma osteoporotic fracture and subsequent fracture in men and women. JAMA.

[3] Cummings SR, Melton LJ (2002). Epidemiology and outcomes of osteoporotic fractures. Lancet Lond Engl.

[4] Baker NL, Cook MN, Arrighi HM, Bullock R (2011). Hip fracture risk and subsequent mortality among Alzheimer’s disease patients in the United Kingdom, 1988-2007. Age Ageing.

[5] Buchner DM, Larson EB (1987). Falls and fractures in patients with Alzheimer-type dementia. JAMA.

[6] Center JR, Nguyen TV, Schneider D, Sambrook PN, Eisman JA (1999). Mortality after all major types of osteoporotic fracture in men and women: an observational study. Lancet Lond Engl.

[7] Drevet S, Bornu B-JC, Boudissa M, Bioteau C, Mazière S, Merloz P (2019). One-year mortality after a hip fracture: prospective study of a cohort of patients aged over 75 years old. Geriatr Psychol Neuropsychiatr Vieil.

[8] Haentjens P, Magaziner J, Colón-Emeric CS, Vanderschueren D, Milisen K, Velkeniers B (2010). Meta-analysis: excess mortality after hip fracture among older women and men. Ann Intern Med.

[9] Pande I, Scott DL, O’Neill TW, Pritchard C, Woolf AD, Davis MJ (2006). Quality of life, morbidity, and mortality after low trauma hip fracture in men. Ann Rheum Dis.

[10] Orive M, Aguirre U, García-Gutiérrez S, Las Hayas C, Bilbao A, González N (2015). Changes in health-related quality of life and activities of daily living after hip fracture because of a fall in elderly patients: a prospective cohort study. Int J Clin Pract.

[11] Haute Autorité de Santé (2017). Note méthodologique et de synthèse documentaire : Orthogériatrie et fracture de la hanche.

[12] American Geriatrics Society (1998). The management of chronic pain in older persons : AGS panel on chronic pain in older persons. Geriatrics..

[13] Aubrun F, Nouette-Gaulain K, Fletcher D, Belbachir A, Beloeil H, Carles M (2019). Revision of expert panel’s guidelines on postoperative pain management. Anaesth Crit Care Pain Med.

[14] Lynch EP, Lazor MA, Gellis JE, Orav J, Goldman L, Marcantonio ER (1998). The impact of postoperative pain on the development of postoperative delirium. Anesth Analg.

[15] Sanzone AG (2016). Current Challenges in Pain Management in Hip Fracture Patients. J Orthop Trauma.

[16] Morrison RS, Magaziner J, McLaughlin MA, Orosz G, Silberzweig SB, Koval KJ (2003). The impact of post-operative pain on outcomes following hip fracture. Pain.

[17] Shyu Y-IL, Chen M-L, Chen M-C, Wu C-C, Su J-Y (2009). Postoperative pain and its impact on quality of life for hip-fractured older people over 12 months after hospital discharge. J Clin Nurs.

[18] Beloeil H, Sulpice L (2016). Peri-operative pain and its consequences. J Visc Surg.

[19] Orosz GM, Magaziner J, Hannan EL, Morrison RS, Koval K, Gilbert M (2004). Association of timing of surgery for hip fracture and patient outcomes. JAMA.

[20] Noll E, Pottecher J, Diemunsch P (2020). Anesthésie pour fracture de l’extrémité supérieure du fémur. Anesth Réanimation.

[21] Oliver D, Griffiths R, Roche J, Sahota O. Hip fracture. BMJ Clin Evid. 2010;2010. PMC2907602 21726483

[22] Haute Autorité de Santé (2000). Évaluation et prise en charge thérapeutique de la douleur chez les personnes âgées ayant des troubles de la communication verbale.

[23] Kelley AS, Siegler EL, Reid MC (2008). Pitfalls and recommendations regarding the Management of Acute Pain among Hospitalized Patients with dementia: table 1. Pain Med.

[24] Ferrell BA, Ferrell BR, Rivera L (1995). Pain in cognitively impaired nursing home patients. J Pain Symptom Manag.

[25] Moschinski K, Kuske S, Andrich S, Stephan A, Gnass I, Sirsch E (2017). Drug-based pain management for people with dementia after hip or pelvic fractures: a systematic review. BMC Geriatr.

[26] Jensen-Dahm C, Palm H, Gasse C, Dahl JB, Waldemar G (2016). Postoperative treatment of pain after hip fracture in elderly patients with dementia. Dement Geriatr Cogn Disord.

[27] Morrison RS, Siu AL (2000). A comparison of pain and its treatment in advanced dementia and cognitively intact patients with hip fracture. J Pain Symptom Manag.

[28] Adunsky A, Levy R, Mizrahi E, Arad M (2002). Exposure to opioid analgesia in cognitively impaired and delirious elderly hip fracture patients. Arch Gerontol Geriatr.

[29] Feldt KS, Ryden MB, Miles S (1998). Treatment of pain in cognitively impaired compared with cognitively intact older patients with hip-fracture. J Am Geriatr Soc.

[30] Knotkova H, Fine PG, Portenoy RK (2009). Opioid rotation: the science and the limitations of the equianalgesic dose table. J Pain Symptom Manag.

[31] Inouye SK, van Dyck CH, Alessi CA, Balkin S, Siegal AP, Horwitz RI (1990). Clarifying confusion: the confusion assessment method. A new method for detection of delirium. Ann Intern Med.

[32] Katz S, Ford AB, Moskowitz RW, Jackson BA, Jaffe MW (1963). Studies of illness in the aged: the index of ADL: a standardized measure of biological and psychosocial function. JAMA..

[33] Lawton MP, Brody EM (1969). Assessment of older people: self-maintaining and instrumental activities of daily living. Gerontologist.

[34] Miller MD, Paradis CF, Houck PR, Mazumdar S, Stack JA, Rifai AH (1992). Rating chronic medical illness burden in geropsychiatric practice and research: application of the cumulative illness rating scale. Psychiatry Res.

[35] Quan H, Li B, Couris CM, Fushimi K, Graham P, Hider P (2011). Updating and validating the Charlson comorbidity index and score for risk adjustment in hospital discharge abstracts using data from 6 countries. Am J Epidemiol.

[36] Mayhew D, Mendonca V, Murthy BVS (2019). A review of ASA physical status - historical perspectives and modern developments. Anaesthesia.

[37] Prestmo A, Hagen G, Sletvold O, Helbostad JL, Thingstad P, Taraldsen K (2015). Comprehensive geriatric care for patients with hip fractures: a prospective, randomised, controlled trial. Lancet Lond Engl.

[38] Harada CN, Natelson Love MC, Triebel KL (2013). Normal cognitive aging. Clin Geriatr Med.

[39] Bruce AJ, Ritchie CW, Blizard R, Lai R, Raven P (2007). The incidence of delirium associated with orthopedic surgery: a meta-analytic review. Int Psychogeriatr.

[40] Pfitzenmeyer P, Musat A, Lenfant L, Turcu A, Musat A (2001). Postoperative cognitive disorders in the elderly. Presse Medicale Paris Fr 1983.

[41] Bliemel C, Lechler P, Oberkircher L, Colcuc C, Balzer-Geldsetzer M, Dodel R (2015). Effect of preexisting cognitive impairment on in-patient treatment and discharge management among elderly patients with hip fractures. Dement Geriatr Cogn Disord.

[42] Dentino A, Medina R, Steinberg E (2017). Pain in the elderly: identification, evaluation, and Management of Older Adults with pain complaints and pain-related symptoms. Prim Care sept.

[43] Rat P, Jouve E, Pickering G, Donnarel L, Nguyen L, Michel M (2011). Validation of an acute pain-behavior scale for older persons with inability to communicate verbally: Algoplus. Eur J Pain Lond Engl.

[44] Morrison RS, Magaziner J, Gilbert M, Koval KJ, McLaughlin MA, Orosz G (2003). Relationship between pain and opioid analgesics on the development of delirium following hip fracture. J Gerontol A Biol Sci Med Sci.

[45] Bouchon JP (1984). 1+2+3 ou comment tenter d’être efficace en gériatrie. Rev Prat.

[46] Pioli G, Bendini C, Giusti A, Pignedoli P, Cappa M, Iotti E (2019). Surgical delay is a risk factor of delirium in hip fracture patients with mild-moderate cognitive impairment. Aging Clin Exp Res.

[47] Schor JD, Levkoff SE, Lipsitz LA, Reilly CH, Cleary PD, Rowe JW (1992). Risk factors for delirium in hospitalized elderly. JAMA.

[48] Hajjar ER, Hanlon JT, Artz MB, Lindblad CI, Pieper CF, Sloane RJ (2003). Adverse drug reaction risk factors in older outpatients. Am J Geriatr Pharmacother.

[49] Crocq MA, Guelfi JD, Boyer P, Pull CB, Pull MC, American Psychiatric Association (2015). DSM-5: diagnostic and statistical manual of mental disorders, 5e edition.

